# Chagas Disease in a Domestic Transmission Cycle in Southern Texas, USA

**DOI:** 10.3201/eid0901.020217

**Published:** 2003-01

**Authors:** Charles B. Beard, Greg Pye, Frank J. Steurer, Ray Rodriguez, Richard Campman, A. Townsend Peterson, Janine Ramsey, Robert A. Wirtz, Laura E. Robinson

**Affiliations:** * Centers for Disease Control and Prevention, Atlanta, Georgia, USA; † Texas Department of Health, Harlingen, Texas, USA; ‡ Cameron County Health Department, San Benito, Texas, USA; § Natural History Museum, University of Kansas, Lawrence, Kansas, USA; ¶ Centro de Investigaciones sobre Enfermedades Infecciosas, Instituto Nacional de Salud Pública Cuernavaca, Morelos, México

**Keywords:** Chagas disease, *Trypanosoma cruzi*, dog, Texas, transmission cycle, dispatch

## Abstract

After three dogs died from acute Chagas cardiomyopathy at one location, an investigation was conducted of the home, garage, and grounds of the owner. A serologic study was conducted on stray dogs, and an ecologic niche model was developed to predict areas where the vector *Tryiatoma gerstaeckeri* might be expected.

## The Study

Chagas disease is caused by the parasitic protozoan *Trypanosoma cruzi* and affects an estimated 12 million persons throughout South and Central America and Mexico (*1*,*2*). In the United States, the disease exists almost exclusively as a zoonosis; only five autochthonous insect-borne cases have been reported in humans (*3*). The distribution of Chagas disease in the United States includes approximately the southern half of the country. Twelve species of triatomines are known to occur in the United States, the most important being *T. sanguisuga* in the eastern United States, *T. gerstaeckeri* in the region of Texas and New Mexico, and *T. rubida* and *T. protracta* in Arizona and California (*4*,*5*).

**Figure 1:**
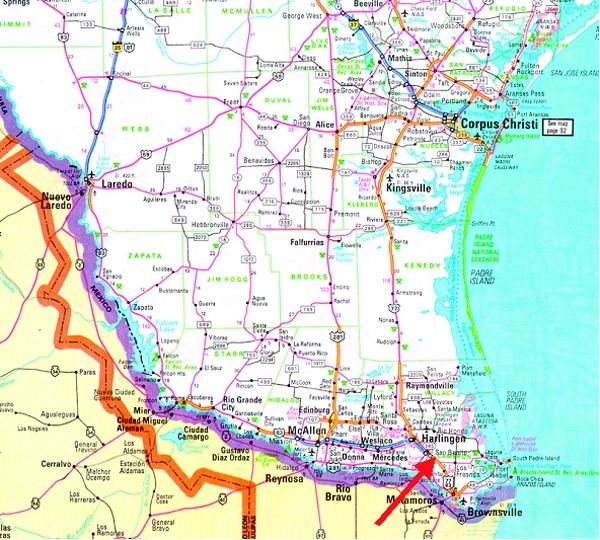
San Benito, Texas, where three dogs died of Chagas disease.

**Figure 2:**
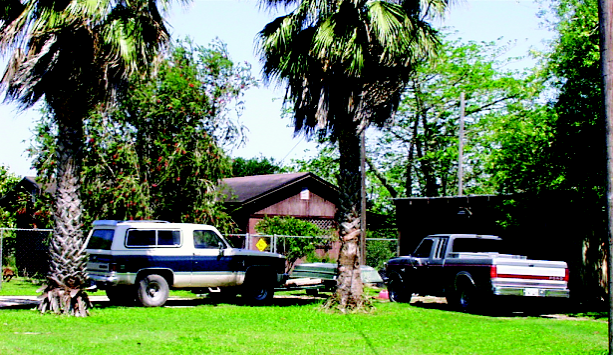
View of location of the three dogs infected with Chagas disease.

In the small community of San Benito, Texas (Figure 1), after three pet dogs died from Chagas cardiomyopathy, personnel from the Texas Department of Health, the Cameron County Health Department, Environmental Health Division, and the Centers for Disease Control and Prevention (CDC) inspected the owner’s home, garage, and grounds for potential triatomine insect vectors (Figure 2). Blood was drawn from four dogs and two persons residing on the property and tested for antibodies to *T. cruzi*. A second site approximately 2 miles away was also inspected and blood drawn from three dogs, one of which had been diagnosed as positive for *T. cruzi* by the original veterinarian. A follow-up serologic survey was conducted to determine the percentage of the stray dogs in Cameron County that would test positive for Chagas disease antibodies. Once a week, samples from stray dogs were shipped to CDC for testing. Each sample was issued an identification number; and information on the animal’s location, sex, age, health condition, and size was recorded. Serum specimens were tested for anti-*T. cruzi* antibodies by indirect immunofluorescence (IIF) (*6*,*7*).

Ecologic niches and potential geographic distributions were modeled by using the Genetic Algorithm for Rule-set Prediction (GARP) (*8**–**10*)*.* In general, the procedure focuses on modeling ecologic niches, the conjunction of ecologic conditions within which a species is able to maintain populations without immigration. Specifically, GARP relates ecologic characteristics of known occurrence points to those of points randomly sampled from the rest of the study region, seeking to develop a series of decision rules that best summarizes those factors associated with the species’ presence. Recently, this method has been used to study the distribution of species complex members and vector-reservoir relationships with respect to Chagas disease (*11*,*12*).

**Figure 3:**
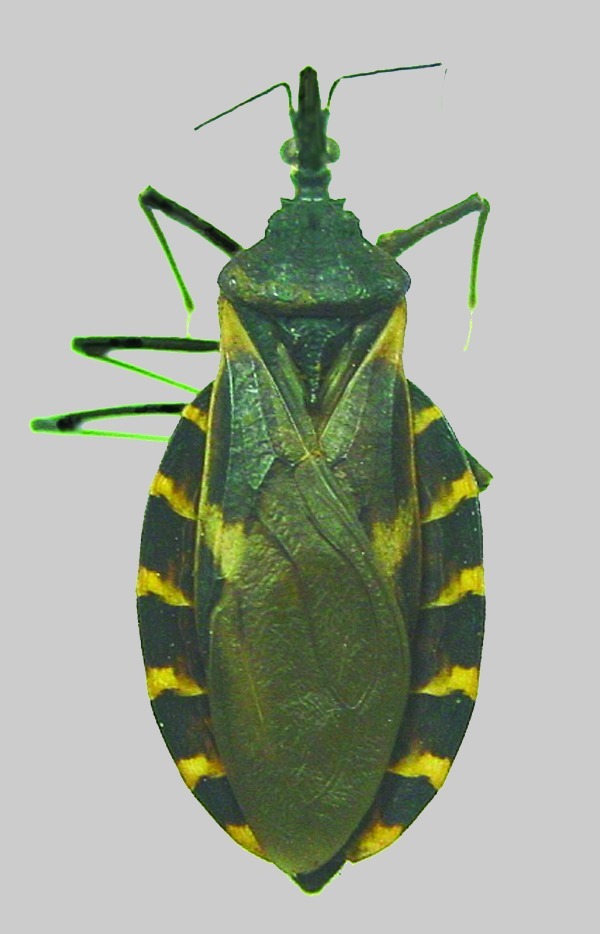
*Trypanosoma cruzi* parasites in hindgut of a field-collected triatomine bugs.

**Figure 4:**
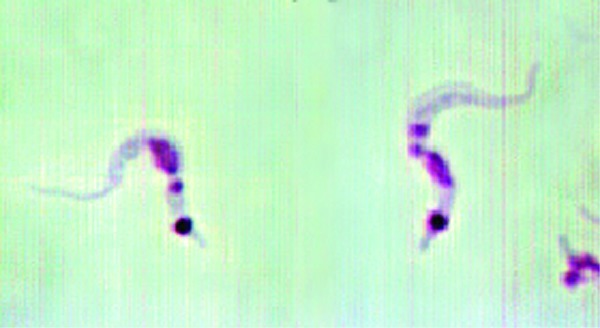
*Triatoma gerstaeckeri* collected at the locality.

**Figure 5:**
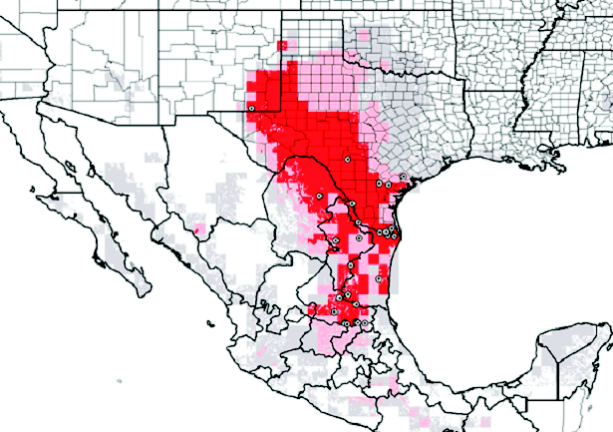
Genetic Algorithm for Rule-set Prediction-generated ecologic niche model, predicting distribution of *Triatoma gerstaeckeri*. Small circles show actual collection sites. Area in dark red is where high certainty exists for the specific niche of the species. The area in light red is the zone of moderate certainty, and the area in gray is for low certainty.

Inspection of the residence where the three dogs lived indicated a substantial infestation with the triatomine species *T. gerstaeckeri* (Figure 3). Triatomines were collected under cement slabs of a backyard patio adjacent to the house and from a garage located approximately 75 feet from the home (Figure 2). Of 31 live triatomines collected, including adults of both sexes and immature stages (i.e., two fifth-instar nymphs), 24 contained *T. cruzi*-like parasites in their hindgut (Figure 4). Cultures were established from triatomine urine collected from insects that were fed in the laboratory and placed in 1.5-mL microcentrifuge tubes. Approximately 50 µL of clear urine was injected into Novy, Nicolle, & MacNeal medium culture (*13*). The cultures were positive for parasites confirmed to be *T. cruzi*, on the basis of morphologic criteria. Inspection of the second residence failed to indicate a bug infestation; however, the pet owner recalled frequently observing both rats (*Rattus* spp.) and opossums (*Didelphis virginiana*) on the premises. At the first site, three of the four dogs tested positive for *T. cruzi*, with titers ranging from 1:128 to 1:256. Neither of the two persons had positive antibody titers against *T. cruzi*. At the second site, only the previously diagnosed dog tested positive, with a titer of 1:256. The other two dogs tested negative, as did the pet owner. Serum samples from stray dogs from Cameron County, Texas, were tested for anti–*T. cruzi* antibodies. Of 375 dogs tested, 28 (7.5%) were positive by IIF, with titers ranging from 1:32 to 1:512. The sensitivity of this test in humans is 98.8% (pers. comm., Patricia P. Wilkins, Division of Parasitic Diseases, CDC). Because of the low specificity of serologic tests for distinguishing *T. cruzi* from *Leishmania* spp., all positive samples were tested for antibodies to *L. donovani*. A low level of cross-reactivity was observed in 17 of the 28 samples. In each case, however, the titer was 1–2 dilutions less than the titer to *T. cruzi*, indicating a primary response to *T. cruzi* rather than to *Leishmania* spp. Ecologic niche models for *T. gerstaeckeri* were developed by using GARP, based on published and unpublished collection records from Mexico and the southwestern United States. The model predicted a distribution for this species that extends from central Mexico, through central Texas, the Texas panhandle, into northern Texas and southeastern New Mexico (Figure 5).

## Conclusions

*Triatoma gerstaeckeri* is considered a sylvatic species, most frequently associated with pack rat (*Neotoma* spp.) burrows (*4*). Although individual triatome insects occasionally invade domestic dwellings throughout the southwestern United States and Mexico (*4*,*5*,*14*), this species has not been reported to colonize these habitats. In this investigation, colonization appears to have occurred, based on the observation of large numbers of bugs, including ones in immature stages. In the Chagas disease–endemic regions of South and Central America, the primary risk for insect transmission to humans is related to the efficiency with which local vector species can invade and colonize homes, resulting in a domestic transmission cycle for what is otherwise exclusively a zoonotic disease in the southern United States. In disease-endemic countries, higher house infestation rates generally result in a higher risk of transmission. At the first site in south Texas, six dogs either died or tested positive for *T. cruzi,* and 24 of 31 bugs contained hindgut trypanosomes. These observations demonstrate the existence of a domestic transmission cycle for an insect species that is typically considered a zoonotic vector. Whether this observation represents an isolated case or actually occurs more frequently but remains unrecognized, indicating an emerging public health problem, remains to be determined. The serologic results in stray dogs are very similar to those reported in previous studies from the region, suggesting that the disease is stably maintained in this reservoir host (*15*,*16*). The distributional predictions based on GARP models indicate a potentially broad distribution for this species and suggest additional areas of risk beyond those previously reported (*14*), should this problem become of greater public health concern.
